# Chemical closed-loop recycling of polymers realized by monomer design

**DOI:** 10.1016/j.fmre.2024.05.015

**Published:** 2024-06-06

**Authors:** Wuchao Zhao, Jianghua He, Yuetao Zhang

**Affiliations:** State Key Laboratory of Supramolecular Structure and Materials, College of Chemistry, Jilin University, Changchun 130012, China

**Keywords:** Chemical recycling, Monomer design, Recyclable polymers, Selective depolymerization, Circular plastic economy

## Abstract

The development of modern society is closely related to polymer materials. However, the improper disposal of the polymer wastes not only squanders resources but also intensifies the environmental issues, despite that energy recovery, physical recycling and chemical recycling pathways have been developed to tackle the recycle and reuse of polymers. Among them, chemical recycling is considered as the most pivotal solution, as it can depolymerize the polymer wastes back to monomers, which then repolymerize into polymer materials. Recently, remarkable progress has been made in the development of chemically recyclable polymers through monomer design to shift “polymerization-depolymerization” equilibrium to realize the selective depolymerization of the polymers into monomers, and to achieve chemical recycling closed-loop. This article reviews the closed-loop polymers such as polyesters, polycarbonates, sulfur-containing polymers, vinyl monomer-based polymers as well as other types of polymers. Moreover, the challenges and prospects in this field are also discussed.

## Graphical abstract

**Figure:**
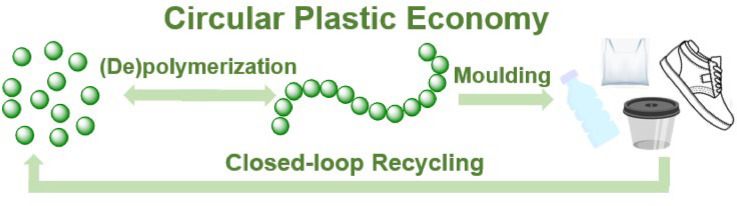

## 1 Introduction

Due to their inexpensive, long-lifetime, and lightweight features, polymers have become an indispensable necessity in modern society. The global demand for polymers has surged dramatically, escalating from 1.65 million tons in 1950 to a staggering 400 million tons presently, and continues to rise at an annual growth rate of 3%–4% [[1], [2], [3], [4], [5]]. Plastics manufacturing is expected to account for 20% of global oil consumption; however, improper disposal of the end-of-life plastics not only causes the waste of resources, but also aggravates environmental problems. The current plastic waste management mainly includes the landfill, incineration, and recycling methods. Approximately, 40% of waste plastics end up in landfills, 14% are incinerated for energy recovery, another 14% are recycled, while the remaining 32% are eventually discarded in the environment, resulting in severe ecological contamination. To solve these issues, efficient recycling of polymeric wastes is essentially important [6].

Various pathways including energy recovery, physical recycling, and chemical recycling have been developed for polymer recycling [[7], [8], [9], [10]]. The simplest one is energy recovery, in which the polymer wastes are incinerated to harness energy. However, this process generates toxic and harmful substances, rendering it the least valuable approach in terms of environmental impact. Next is physical recycling, where the plastic wastes are collected, cleaned, and directly reprocessed into products. Typically, this method often yields the products with deteriorated properties and reduced value due to the presence of impurities, thus being considered as less effective recycling. Chemical recycling can be divided into two distinct processes: 1) transforming the polymers into new monomers different from the original ones, and 2) depolymerizing the polymers back to the virgin monomers and then repolymerizing these monomers into polymers with the identical properties, thus achieving a closed-loop recycling. Different from the aforementioned recycling methods, the closed-loop recycling maintains the properties of the plastic, furnishing the most desirable form of recycling. Although solvolysis, enzymatic hydrolysis and catalytic recovery have been successfully industrialized, the closed-loop recycling still faces great challenges. At present, polymers are typically obtained with enhanced material properties and extended service life by increasing chemical inertness, which plays obstacles in realizing chemical recycling, such as poor selectivity, low yields, and high energy consumption.

With the progress of polymer science, increasing attention has been focused on the development of the next-generation of chemically closed-loop recyclable polymers [1,[11], [12], [13], [14], [15]]. For polymer chemists, the focus on both the performance and degradation of materials is crucially important. However, both the intrinsic contradiction between the polymerization and depolymerization, and the durability of polymers, are major obstacles that hinder the development of closed-loop recycling of polymers. To this end, a strategy based on monomer design becomes pivotal, which not only enables the facile synthesis of polymers for practical application, but also makes polymers recyclable. Although there have been several reviews focusing on the downcycling, upcycling, and closed-loop recycling of polymers, the realm of polymer recycling is evolving rapidly [[16], [17], [18], [19], [20]]. Particularly, this review will spotlight the progress in the development of monomers aimed at building high-performance closed-loop recyclable polymers, including polyesters, polycarbonates, sulfur-containing polymers, polycyclic olefins, polar vinyl monomer polymers, and other types.

## 2 The general principles of monomer design

**Fig. 1:**
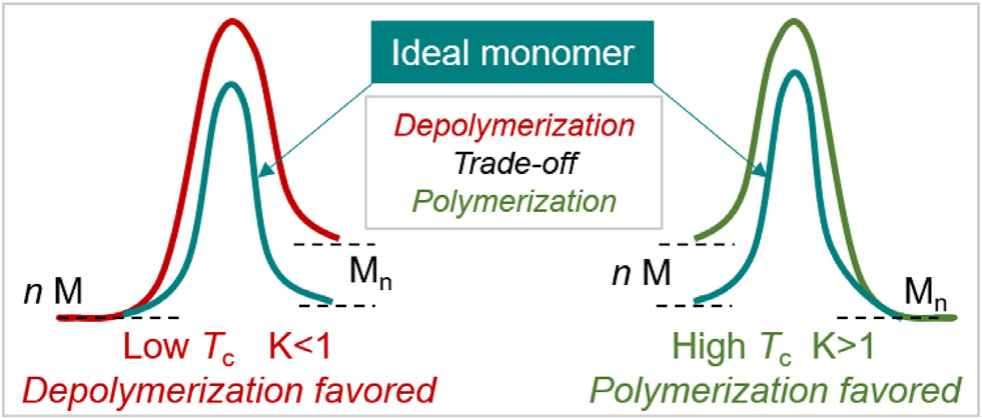
**Monomer design principles of closed-loop recyclable polymers based on thermodynamic and kinetic considerations**.

Polymerization reactions require the Gibbs free energy change (ΔG) to be less than 0, and the ΔG during the polymerization process is closely related to the enthalpy change (ΔH), entropy change (ΔS), and temperature [21]. The majority of polymerization are enthalpy-driven reactions (ΔH < 0) (e.g., ring-opening polymerization (ROP) of small to medium-ring lactones), because ΔS < 0 during the polymerization process. According to ΔG = ΔH-TΔS, when polymerization and depolymerization reactions reach equilibrium (ΔG = 0, equilibrium constant *K*=1), the reaction temperature is the ceiling temperature (*T*_c_), at which a temperature lower than *T*_c_ (*K* > 1) favors the generation of polymers with high molecular weight (Fig. 1), while a temperature higher than *T*_c_ (*K* < 1) favors the depolymerization of polymers back to monomers. The *T*_c_ of polymerization is not only related to monomer structure but also to the polymerization conditions. By changing reaction conditions, the "polymerization-depolymerization" equilibrium can be disrupted. For example, precipitating polymers from the system can increase the monomer conversion, while removing monomers from depolymerization through processes like sublimation or distillation can increase the depolymerization yield. It should be emphasized that depolymerization does not necessarily occur when the temperature is higher than *T*_c_, and depolymerization requires overcoming a certain energy barrier, so the service temperature of the polymer can be higher than its *T*_c_. In summary, adjusting the *T*_c_ of polymerization reactions through monomer structure design can regulate the "polymerization-depolymerization" equilibrium. Developing an "ideal monomer" that approaches thermodynamic equilibrium under mild conditions provides foundation for constructing closed-loop recycling of polymers. For cyclic monomers, the *T*_c_ of chain-growth polymerization can be effectively regulated by introducing the side groups, fused rings, or heteroatoms into the cyclic frameworks to achieve the closed-loop recycling. However, for low-*T*_c_ vinyl polymers, the main concern is on how to initiate depolymerization, rather than on monomer design.

## 3 Chemical closed-loop recycling of polyesters

Polyesters with ester functional groups in the main chain are biodegradable and sustainable. Their versatility is demonstrated by the ability to adjust thermal and mechanical properties through the modification of the length and substituents of the main chain [22,23], which made them to be a promising substitute for the petroleum-based polymers, in the fields of packaging, medicine, electronics, and construction. Polyesters, prepared from ROP, offer adjustable polymerization-depolymerization equilibrium by altering the ring size and substituents of cyclic monomers [12,24], which enables the direct depolymerization of polyesters back to monomers under mild conditions, highlighting their potential chemical recyclability. Progress has been made in the design of lactones for the closed-loop recycling in recent years, which will be discussed on the basis of the ring sizes.

### 3.1 Four-membered cyclic esters

Polyhydroxyalkanoates (PHAs) are versatile polyesters known for their adjustable properties, biodegradability, and as the sustainable alternatives to non-degradable plastics [[25], [26], [27]]. Despite their promise in addressing the plastic problem, PHAs face significant challenges, including limited melt processability, mechanical brittleness and lack of recyclability. Overcoming these challenges, especially improving recyclability, is crucial for PHAs to contribute effectively to a closed-loop plastic economy.

**Fig. 2:**
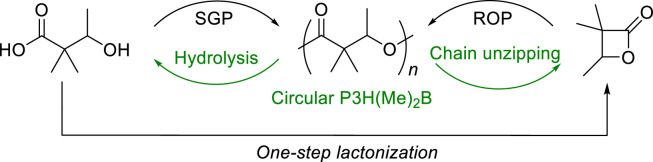
**Chemical circularity of P3H(Me)**_**2**_**B realized by the closing of both the hydroxyacid SGP, hydrolysis and lactone ROP, and chain-unzipping loops by base**.

Recently, Chen developed a PHA synthetic platform that addressed the thermal instability of PHAs by replacing the α-hydrogen in PHA repeat units with alkyl groups, thereby reducing the possible *cis*-elimination commonly occurring during thermal degradation (Fig. 2) [28]. This led to the production of poly (3‑hydroxy-2,2-dimethylbutyrate) [P3H(Me)_2_B], with significantly enhanced thermodynamic properties. P3H(Me)_2_B is semicrystalline with a high melting temperature (*T*_m_) in the range of 167–243 °C, and shows excellent thermal stability and melt processability ranging between 314–335 °C. P3H(Me)_2_B exhibited toughness with strain over 200% and can be chemically recycled. Its starting monomer α, α-dimethyl-β-butyrolactone [(Me)_2_BL] can be used for chain growth ROP, or 3-hydroxy-2,2-dimethylbutyric acid [3H(Me)_2_BA] can be used for step growth polycondensation (SGP), thus achieving closed-loop chemical recycling. This method can rapidly produce P3H(Me)_2_B with low to high molar mass (number-average molecular weight (*M*_n_) up to 554 kg/mol) while maintaining narrow molecular weight distribution (*Ð* as small as 1.02), and near 100% yield, with the catalysis of *^t^*Bu-P_4_.

### 3.2 Five-membered cyclic esters

Five-membered cyclic esters, such as γ-butyrolactone (γBL), are relatively stable, and commonly referred as ‘non-polymerizable’ due to their low strain energy. For a long time, γBL can only be polymerized under extremely harsh conditions (20,000 atm, 160 °C). However, these polymers were produced with very low molecular weights (about 5.0 kg/mol), which limits their further applications [[29], [30], [31]].

**Fig. 3:**
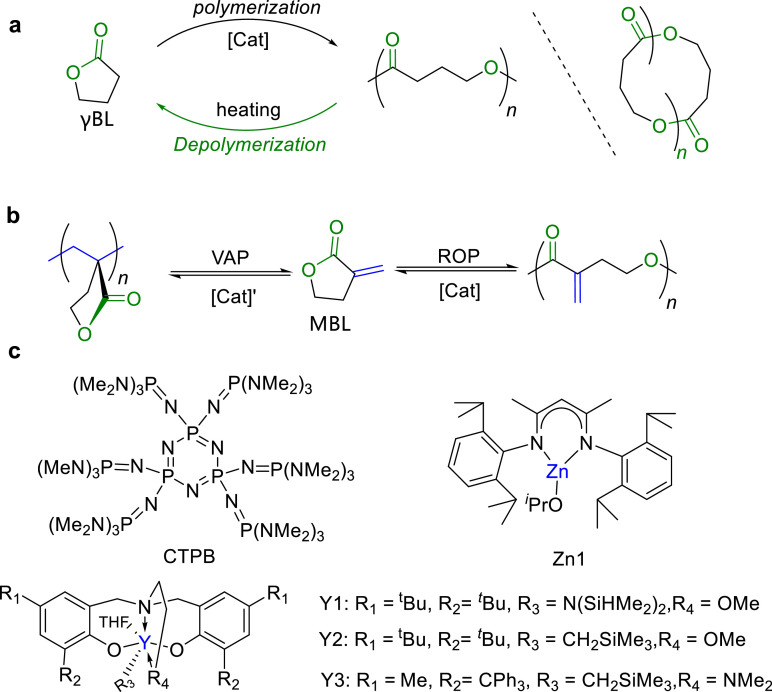
Chemical recyclable (a) γBL and (b) MBL. (c) Catalysts used for the ROP of γBL and MBL.

In 2016, Chen et al. realized the ROP of ‘non-polymerizable’ γBL under normal pressure (Fig. 3a) [24]. They re-evaluated the thermodynamic parameters of ROP of γBL. Its Δ*S* is −39.6 J mol^-1^ K^-1^, and Δ*H* is −5.4 kJ mol^-1^, confirming the theoretical feasibility of ROP of γBL. To efficiently polymerize γBL, they proposed two strategies. To minimize entropy loss, in the first strategy, polymerization was conducted at a low temperature (below the *T*_c_ of polymerization) and a high monomer concentration (above the equilibrium monomer concentration). In the second strategy, the reaction conditions were adjusted, such as concentration, solvent, and temperature, such that the monomer concentration [M] exceeded the equilibrium concentration [M]_eq_. This would ensure that polymers either crystallize or precipitate out of the solution during polymerization, thus continuously perturbing the polymerization/depolymerization equilibrium and favoring chain growth. Using the above strategies, Chen et al. successfully realized the ROP of γBL by using La[N(SiMe_3_)_2_]_3_ or Y1 as a catalyst (Fig. 3c). The polymerization was carried out with a starting monomer concentration of 10 mol/L at −40 °C, obtaining up to 90% monomer conversion and polymers with *M*_n_ up to 30.0 kg/mol.

By changing the catalyst to initiator ratios, PγBL can be obtained with either linear or cyclic topologies. The produced PγBL can be depolymerized back to its original monomer simply by heating at 220 °C for linear PγBL and 300 °C for cyclic PγBL, respectively. This process reveals that cyclic PγBL exhibited higher thermal stability compared with its linear counterpart. Then, *^t^*Bu-P_4_ was utilized as a catalyst for ROP of γBL [32]. Since then, numerous catalysts (Fig. 3c) have been reported for ROP of γBL, including cyclic organic base CTPB and dual base/(thio)urea systems, such as *^t^*Bu-P_4_/thioureas, alkoxides/ureas, and CTPB/ureas [31,[33], [34], [35]]. Zhang and Li separately reported that the molecular weight of PγBL significantly affected its mechanical properties. For example, the stress and strain of PγBL can reach up to 40.4 MPa and 858%, respectively, which is comparable to the poly(4-hydroxybutyrate) produced via biological fermentation.

α-Methylene-γ-butyrolactone (MBL) is a bifunctional monomer derived from biomass, featuring a highly reactive exocyclic C=C bond and a stable five-membered γ-butyrolactone ring (Fig. 3b). The vinyl-addition polymerization (VAP) of exocyclic C=C bond furnished the polymers with a high glass transition temperature (*T*_g_) ∼ 195 °C, making it a potential alternative to PMMA [[36], [37], [38]]. However, ROP of MBL is challenging due to the strained nature of five-membered ring and the *cis*-structure of the ester group and exocyclic C=C bond, which makes VAP more competitive.

Based on these successful ROP of γBL, Chen et al. reported ROP of MBL under the condition of [M]_0_ = 5.0 M, and temperature = −60 °C by using La[N(SiMe_3_)_2_]_3_/ROH = 1/3 or Y1 as a catalyst, furnishing polyesters P(MBL)_ROP_ with *M*_n_ up to 21.0 kg/mol, and *Ð* = 1.46 [39]. By heating a solution of P(MBL)_ROP_ in DMSO in the presence of La[N(SiMe_3_)_2_]_3_ and water at 100 °C or 130 °C for 1 h, or 60 °C for 24 h, MBL was cleanly recovered in a quantitative yield, confirming the closed-loop recycling feature of P(MBL)_ROP_. Subsequently, Li and Lu reported the CTPB/ureas catalyzed ROP of MBL and obtained 45% monomer conversion after 4 h, furnishing P(MBL)_ROP_ with *M*_n_ up to 6.7 kg/mol [40]. Similar as other polyesters, P(MBL)_ROP_ exhibited semicrystalline property with *T*_m_ ∼ 44 °C.

**Fig. 4:**
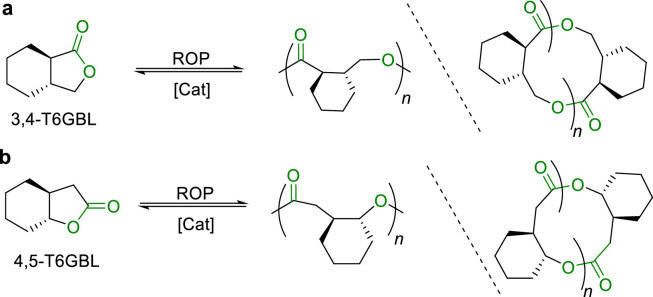
**Chemically recyclable (a) 3,4-T6GBL and (b) 4,5-T6GBL**.

Although the polyesters obtained from ROP of γBL and MBL can be easily and selectively depolymerized back into monomers, low-temperature polymerizations were typically required and the polymers were often produced with insufficient physical robustness and mechanical strength for practical applications. To solve these issues, Chen introduced a *trans*-cyclohexyl ring to the α and β positions of γBL [41], which significantly increased the ring strain in the five-membered ring, leading to the production of a new monomer, trans hexahydro-2(3H)-benzofuranone (3,4-T6GBL) (Fig. 4a), noted for its high polymerization activity. The ROP of 3,4-T6GBL exhibited a Δ*S* of −72.0 J mol^-1^ K^-1^, and Δ*H* of −20 kJ/mol, which allowed for its bulk polymerization at RT by using La[N(SiMe_3_)_2_]_3_, Y1 or Zn1 as a catalyst, furnishing polymers with *M*_n_ up to 1110 kg/mol. The isotactic P(3,4-T6GBL) showed a *T*_m_ of 127 °C. Through the stereocomplex crystallization of isotactic P(3,4-T6GBL) with opposite configurations, the *T*_m_ can be further enhanced to 203 °C. More importantly, P(3,4-T6GBL) can be quantitatively recycled back to its original monomer through thermolysis at ≥ 300 °C for 24 h or chemolysis by ZnCl_2_ at 120 °C.

On the basis of the above-described advancements, Chen further developed stereoselective ROP of *rac*-3,4-T6GBL by modifying the sidearm of Y-based catalyst ligands [42]. Heterotactic P(3,4-T6GBL) with *P*_r_ up to 0.75, and isotactic P(3,4-T6GBL) with *P*_m_ up to 0.95 can be prepared by different Y-based catalysts (Fig. 3c), respectively. Moreover, they successfully achieved living coordination ROP of 4,5-*trans*-cyclohexyl-fused γ-butyrolactone (4,5-T6GBL) at RT by La[N(SiMe_3_)_2_]_3_ or base/thiourea system (Fig. 4b) [43,44], affording P(4,5-T6GBL) with *M*_n_ up to 215 kg/mol, which is a notable improvement from the oligomers obtained previously. P(4,5-T6GBL) also demonstrated closed-loop chemical recycling *via* chemolysis.

**Fig. 5:**
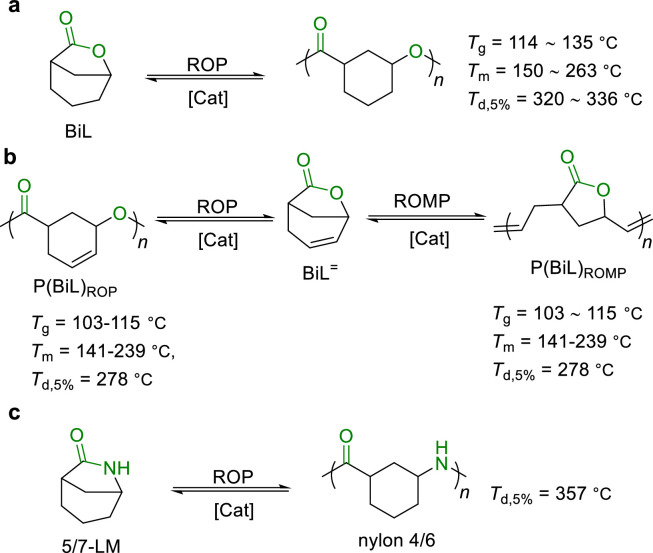
**Chemical recycling of (a) hybrid monomer BiL, (b) hybrid monomer BiL**^**=**^**and (c) hybrid monomer 5/7-LM**.

To enhance the mechanical properties of the polymers while maintaining high polymerizability and depolymerizability, Chen et al. developed a hybrid monomer design strategy [12,45]. This approach utilized a bicyclic lactone (BiL) monomer (Fig. 5a) to synergistically combine a high *T*_c_ sub-structure for high polymerizability and mechanical properties with a low *T*_c_ sub-structure for high depolymerizability and recyclability. The produced PBiL exhibited adjustable *T*_g_ (114–135 °C), *T*_m_ (150–263 °C) and *T*_d,5__%_ (320–336 °C), surpassing the properties of PCL or PγBL. The mechanical properties of PBiL can be regulated by altering the microstructure of the polymer chain. Notably, PBiL can be depolymerized back to BiL in quantitative yield with the catalysis of La[N(SiMe_3_)_2_]_3_ at 120 °C.

By using similar hybrid monomer design strategy, Chen reported a bicyclic lactone/olefin (BiL^=^) as bifunctional monomer (Fig. 5b) [46]. This monomer is particularly versatile: the cyclohexene part can proceed ring-opening metathesis polymerization (ROMP), and the lactone part can be polymerized via ROP, offering two distinct classes of polymers P(BiL^=^)_ROMP_ and P(BiL^=^)_ROP_ from the same monomer, respectively. Both polymers exhibited good thermal stability and can be chemically recycled in a closed-loop process under mild conditions, with the help of a catalyst in facilitating the chain unzipping and scission.

Furthermore, Chen combined two parent structures, caprolactam 7-LM (high *T*_c_) and five-membered lactam 5-LM (low *T*_c_), into a hybrid lactam monomer 5/7-LM with a double ring structure (Fig. 5c) [47]. This monomer combines the advantages of both parent structures while eliminating their disadvantages. The corresponding polymer nylon 4/6 showed both good depolymerizability and excellent performance, and 5/7-LM can be copolymerized with 5-LM to furnish hybrid nylon exhibiting excellent optical permeability and chemical recycling. This work showcases the potential of hybrid monomer designs in synthesis of polymers with enhanced properties and recyclability.

### 3.3 Six-membered cyclic esters

δ-Valerolactone (δVL) and the related six-membered cyclic esters have the higher ring strain than that of γBL, which endows them higher *T*_c_, thus higher polymerization activity. Several substituted δVLs have been prepared from biomass and investigated for their polymerization, making them promising candidates for synthesis of chemically recyclable polymers.

**Fig. 6:**
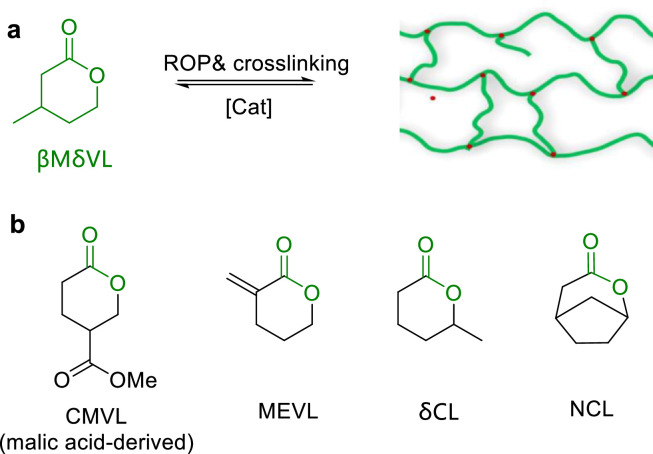
**Chemical recycling of (a) crosslinked P(βMδVL) and (b) CMVL, MEVL, δCL and NCL**.

In 2014, Zhang and Hillmyer et al. demonstrated a completely biosynthetic pathway for production of β-methyl-δ-valerolactone (βMδVL) from glucose (Fig. 6a) [48]. Following this, Hillmyer explored the copolymerization of βMδVL with bis(six-membered cyclic carbonate) and preparation of cross-linking P(βMδVL) using a free-radical generator [49]. This process yielded the cross-linked elastomers with impressive tensile strength (up to 12 MPa) and elongation at break (up to 2000%). These P(βMδVL) based elastomers can be chemically recycled, furnishing monomer with high purity (> 91%) and yield (> 93%). Later, βMδVL was also used for preparation of chemically recycled polyurethane, demonstrating that βMδVL is an attractive feedstock for synthesis of sustainable polymeric materials [50].

In 2018, Hoye et al. employed 4-carbomethoxyvalerolactone (CMVL) derived from malic acid for synthesis of poly(4-carbomethoxyvalerolactone) (PCMVL) (Fig. 6b) [51], furnishing a high monomer conversion of 98.3% and semicrystalline PCMVL with two distinct melting points at 68 °C and 86 °C, respectively. Notably, this polyester can be chemically recycled back to CMVL in 87% yield, by using Sn(Oct)_2_ as a catalyst. In 2020, they reported 4-carboalkoxyvalerolactones (CRVLs) and discovered that alkyl side chains have significant influences on the properties and degradation rates of these polyesters [52].

In 2019, Xu and coworker reported chemoselective ROP of bifunctional monomer α-methylene-δ-valerolactone (MEVL) by N-heterocyclic carbene (NHC) and discovered that solvents can significantly influence the resulting polyesters (Fig. 6b) [53]. With the catalysis of Sn(Oct)_2_, these polyesters can be completely converted back to the virgin monomers through thermochemical processes. Later, Li and Shen further investigated the ROP of MEVL and realized precision control over the polymerization [54].

In 2022, Li and Shen employed the base/urea binary catalyst system to achieve controlled ROP of δ-caprolactone (δCL) derived from 5-hydroxymethylfurfural (Fig. 6b) [55]. Both MeOK and *^t^*Bu-P_2_ showed well control over the molar weight (*M*_n_ up to 101 kg/mol) and molecular weight distribution (*Ð* as low as 1.07) of the produced polymers. Through the sequential ROP of δCL and l-lactide (LLA), well-defined tri-block copolymers PLLA-*b*-PδCL-*b*-PLLA were prepared and showed excellent thermoplastic elastomers (TPEs) properties. PLLA_150_-*b*-PδCL_700_-*b*-PLLA_150_ exhibited high elastic recovery (93.1 ± 0.1%) and tensile strain (755 ± 33%), comparable with the commercial petroleum-based TPEs. Furthermore, PδCL can be quantitatively converted back to δCL in the presence of Sn(Oct)_2_ (bulk, 130 °C). Subsequently, they prepared telechelic PδCL diol precursors to react with diisocyanate for synthesis of thermoplastic polyurethane elastomers, which can be also degraded into δCL with the catalysis of Sn(Oct)_2_ at 180 °C [56].

Rieger reported the ROP of norcamphor lactone (NCL), derived from commercial norcamphor through a one-step Baeyer-Villiger oxidation process (Fig. 6b) [57]. By using ZnEt_2_ as a catalyst, PNCLs with *M*_n_ up to 164 kg/mol were successfully prepared at 110 °C. Interestingly, under thermolysis conditions, PNCLs can be completely depolymerized into the pristine monomer NCL in over 90% yield.

**Fig. 7:**
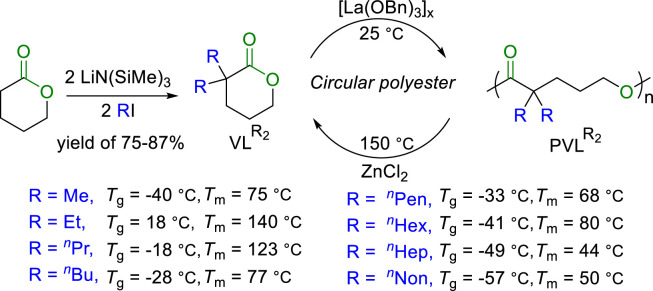
**Synthesis and chemical recycling of**$VL^{R_{2}}$.

Geminal disubstitution in cyclic monomers, such as δVL, was demonstrated to be effective for the enhancement of the polymer recyclability (Fig. 7). Recently, Xu and Chen reported the gem‑α,α-disubstitution of δVL for production of gem‑dialkyl-substituted valerolactones ($VL^{R_{2}}$), which can be polymerized into the gem‑disubstituted polyesters ($\text{PV}L^{R_{2}}$). Such polymers not only exhibited completely chemical recyclability but also thermal, mechanical and transport properties comparable with or superior to the polyethylene [58]. They synthesized eight $VL^{R_{2}}s$ with different gem‑α,α-disubstitution. The corresponding *T*_c_ of the polymers can be regulated in the range of 67∼115 °C. [La(OBn)_3_]_x_ was utilized to catalyze the living polymerization of $VL^{R_{2}}s$, furnishing polymers with *T*_g_s in the range of −57∼18 °C, and *T*_m_s in the range of 44∼140 °C. For comparison purpose, $\text{PV}L^{Pr_{2}}$ was prepared to exhibit mechanic properties (*ε*_B_ = 209 ± 13%; *σ*_B_ = 44 ± 2.6 MPa) superior to LDPE standard in all stress-strain curve area, and higher ultimate strength than HDPE standard.

**Fig. 8:**
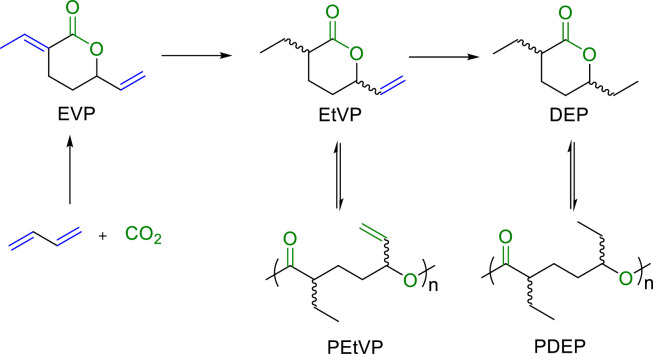
**Synthesis and chemical recycling of EtVP and DEP**.

Carbon dioxide is recognized as an ideal sustainable feedstock for polymer synthesis, offering a path towards achieving carbon neutrality and alleviating the environmental pollution from the polymer wastes. 3-Ethyl-6-vinyltetrahydro-2H-pyran-2-one (EtVP) and hydrogenated 3,6‑diethyl-tetrahydro-2H-pyran-2-one (DEP), derived from the reaction of CO_2_ and butadiene, have been identified for their complete chemical recyclability due to their low *T*_c_s (18.3 and −6 °C at [M]_0_ = 1.0, respectively) (Fig. 8) [[59], [60], [61]]. MeOK was utilized to catalyze the polymerization of EtVP into polyesters with *M*_n_ up to 588 kg/mol and *Ð* = 1.14 at −25 °C, while leaving double bond for further derivation. And PDEP shows pressure-sensitive adhesive (PSA) properties comparable to those of the commercial products. More strikingly, these CO_2_ based polyesters can be quantitatively converted back to their pristine monomers, by using La[N(SiMe_3_)_2_]_3_ as chemolysis catalyst.

**Fig. 9:**
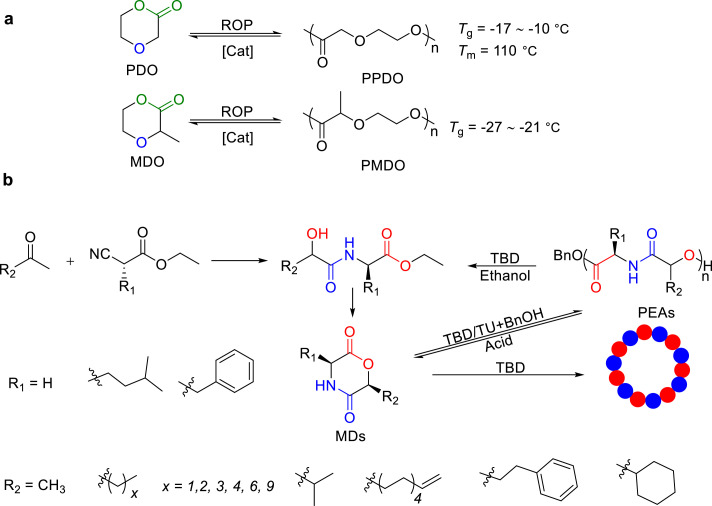
(a) Chemical recycling of PDO and MDO. (b) Synthesis and chemical recycling of MDs.

Beyond polyesters, chemically recyclable poly(ether-ester)s such as poly(*p*-dioxanone) (PPDO) also exhibited superior biocompatibility, degradability, and mechanical robustness, making them highly suitable for biomedical applications (Fig. 9a) [62]. Being derived from six-membered ring of *p*-dioxanone (PDO), the unique structural feature of PPDO enables its degradation back into monomers upon heating (Fig. 9a). In 2021, Li and Shen reported the ROP of 3-methyl-1,4-dioxan-2-one (MDO) by base/urea, affording PMDO, a PDO analogue [63]. In contrast to PPDO, PMDO is amorphous with *T*_g_ ranging from −21 to −27 °C. With the catalysis of 10 mol% 1,8-diazabicyclo[5.4.0]undec‑7-ene (DBU), a complete monomer recovery can be achieved by heating the solution at 140 °C for 6 h, thereby establishing a sustainable closed-loop lifecycle for these materials.

Recently, Li and Du reported the closed-loop recyclable aliphatic poly(ester-amide)s (PEAs) (Fig. 9b) [64]. They utilized 1,5,7-triazabicyclo[4.4.0]dec‑5-ene (TBD) and thiourea as catalysts to achieve living ROP of morpholine-2,5‑dione derivatives (MDs), yielding PEAs with predicted molar masses and narrow molecular weight distribution. Two approaches were adopted for depolymerization: 1) TBD-catalyzed alcoholysis can rapidly and quantitatively depolymerize the polymers back to the original monomers, and 2) amberlyst-15 ion-exchange resin-catalyzed depolymerization quantitatively produced pure monomers. Further investigation was expanded to 6-alkyl-substituted MDs [65]. Tensile testing revealed that the structure of pendant alkyl groups can significantly affect the mechanical properties of PEAs. Notably, PEAs substituted with *n*‑butyl or cyclohexyl groups are brittle, while those with *n*-hexyl or *n*-octyl substituents were ductile plastics.

### 3.4 Seven-membered cyclic esters

Poly(ε-caprolactone) (PCL) was prepared from the ROP of ε-caprolactone (εCL), a typical seven-membered cyclic ester known for its excellent biocompatibility and biodegradability. However, its high *T*_c_ necessitates the high thermal degradation temperature, resulting in low monomer recovery yields. Progress has been made in the employment of catalysts to lower the degradation temperature. Recently, Wang et al. reported that PCL can be depolymerized into εCL in 98% yield by using Mg(HMDS)_2_ as a catalyst [66].

**Fig. 10:**
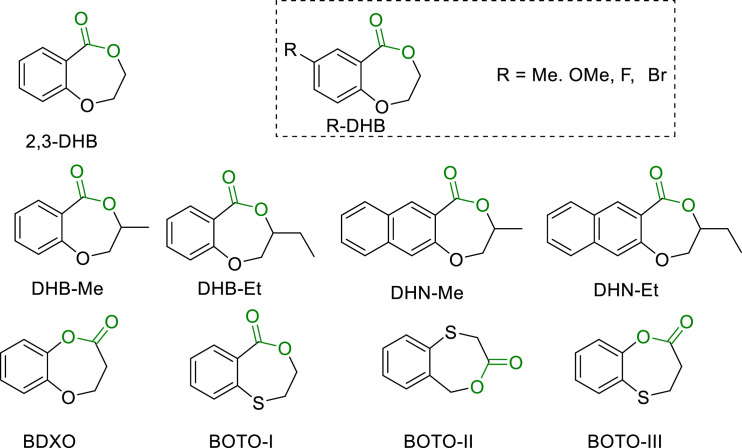
**Structures of chemically recyclable 2,3-DHB, R-DHBs, DHB-Rs, DHN-Rs, BDXO, and BOTO-I-III**.

It is reported that incorporating an aromatic ring into a seven-membered cyclic monomer can reduce its ring tension, thereby improving the polymer's degradability. In 2016, Shaver's group successfully utilized Salene-Al catalyst for the ROP of 2,3-dihydro-5H-1,4-benzodioxepin-5-one (2,3-DHB), producing aromatic polyester P2HEB, the first example that with an aromatic ring in the backbone (Fig. 10) [67]. P2HEB undergoes depolymerization in the presence of enzyme or Salene-Al catalyst. In addition, increasing the temperature and decreasing the concentration can convert the polymers back to the monomers.

Subsequently, Makwana, Lizundia, et al. introduced the substituents to the meta-position of the benzene ring to furnish the polymers P(R-DHB)s with enhanced thermal stability, exhibiting *T*_d_ between 230 and 260 °C (Fig. 10) [68]. Similarly, Zhu and Cai incorporated the substituents into both the benzene and ester rings (DHB-Rs) to produce polymers with even higher thermal stability (335 °C < *T*_d_ < 350 °C) (Fig. 9) [69]. Wang et al. encountered the difficulties in preparing high molecular weight poly(phenolic esters) due to the occurrence of significant transesterification and back-biting reactions during the polymerization and depolymerization of the phenolic ester monomer BDXO (Fig. 9) [70].

In 2021, Li and Du synthesized three benzo-thia-caprolactone isomers (BOTO-I, BOTO-II, and BOTO-III) and obtained semicrystalline materials with slow crystallization rates upon polymerization (Fig. 10) [71]. P(BOTO-I) exhibited the highest *T*_m_ at 146 °C and a *T*_g_ of 59 °C. P(BOTO-I) and P(BOTO-II) maintained stability up to 270 °C, similar as PLA, whereas P(BOTO-III) began decomposing at 180 °C. Tensile testing revealed that P(BOTO-I) possessed a slightly lower Young's modulus but significantly higher elongation than that of the PLLA or its stereocomplex, making it tougher than PLLA. The depolymerization of P(BOTO)s in solution yielded a mixture of monomers and oligomers, whereas bulk thermal depolymerization selectively produced corresponding monomers in high yields.

**Fig. 11:**
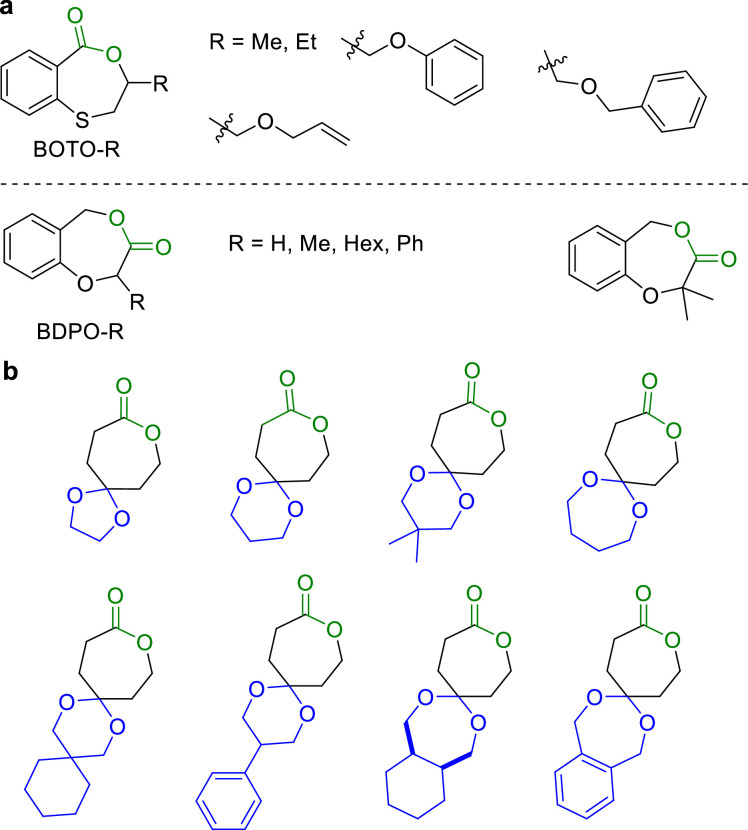
**Structure of the chemical recyclable (a) BOTO-R and (b) Spiral ring lactone monomers based on εCL**.

Despite the promising potentials of these polymers in depolymerization back into monomers, the practical applications were limited by their low crystallinity and mechanical properties. To address this issue, Zhu et al. designed and synthesized a series of aromatic cyclic esters (BOTO-R) from the reaction of thiosalicylic acid and epoxides in one-pot manner (Fig. 11a) [72]. These monomers can be polymerized into polymers exhibiting remarkable thermal stabilities (*T*_d_ > 286 °C). DSC analysis showed that the thermal properties were significantly influenced by the substituents, and no *T*_m_ was observed, indicating the amorphous nature of the polymers. Isotactic polymers produced from the polymerization of chiral monomers were crystalline. Mixing different chiral polymers in a 1:1 ratio resulted in stereo-composite crystallization, substantially enhancing the material's *T*_m_. Tensile tests further confirmed the profound impact of substituent structures on the mechanical properties. Modifying the substituent (i.e. *R* = Me, strong and brittle polymers; σ_B_ = 61.08 MPa, ε_B_ = 4.43%) allowed property regulation, significantly enhancing the toughness when compared with the polymers with R = CH_2_CHCH_2_OCH_2_- (σ_B_ = 20.96 MPa, ε_B_ = 311.6%). Copolymerization between the monomers possessing different substituents further modulated the material's properties, eventually affording aromatic polyesters with mechanical properties comparable to that of isotactic polypropylene. More importantly, they discovered that P(BOTO-R)s can be near quantitatively converted back to monomers in dilute solutions, by using the Zn-based catalyst.

Incorporating an aryl ring into a cyclic monomer often diminishes its polymerization activity due to the conjugation effects between the aromatic ring and the carbonyl group. It remains as a formidable challenge to strike the balance between polymerization efficiency and the performance of the resulting polymers. Zhu et al. accomplished this goal by relocating the ester groups in the aromatic monomers to sever their direct connection to the aromatic ring, thereby enhancing the ROP activity and furnishing high-performance recyclable polyesters. They synthesized a variety of monomers, BDPO-R (Fig. 11a), from the biobased salicylaldehyde with different substituents [73]. These monomers can be polymerized into polymers with *M*_n_ up to 438 kg/mol. The resulting aromatic polyesters exhibited excellent thermal stabilities (*T*_d_ > 275 °C), and their *T*_g_s can be tuned from −1 to 79 °C by altering the substituent structures. More importantly, these polymers can be efficiently and selectively depolymerized back into monomers under both solution and bulk conditions, with an *ee* value exceeding 99%, which allowed the direct recycling from polymerization without further purification. Later, they developed a series of BDPO-based monomers with methoxy‑, bromo‑, and naphthalene-substituents, enhancing the tunability of both the monomers’ activities and polymers’ properties. These monomers maintained high polymerizability and recyclable characteristics of the BDPO system. More recently, they designed a class of spiral ring lactone monomers based on εCL framework (Fig. 11b) [74]. Their polymers demonstrate not only the ability to fully degrade and recycle, but also the tunable properties by altering the cyclic structure. For instance, modifying the ring size or rigidity of the cyclic structure can adjust the crystallinity and mechanical properties of the polymers in a wide range, which would enable the synthesis of either elastomers or tough plastics, offering wide application potential of such polymers.

**Fig. 12:**
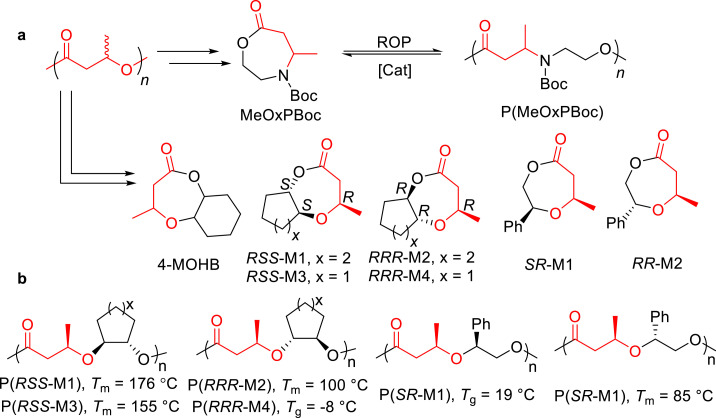
(a) Synthesis and chemical recycling of MeOxPBoc, 4-MOHB and enantiopure ether-ester monomers. (b) Structures of crystalline stereoregular poly(ether-ester).

Crotonic acid (CA), featuring a reactive double bond and a carboxylic group, can be efficiently derived from the pyrolysis of poly(3-hydroxybutyrate) (P3HB) [25]. Li and Shen et al. synthesized the N-heterocyclic lactone MeOxPBoc from CA (Fig. 12a) [75]. Utilizing Sn(Oct)_2_ as a catalyst promoted living ROP of MeOxPBoc, producing amorphous poly(amine-*alt*-ester) P(MeOxPBoc). This polymer can be depolymerized into its original monomer either in trimethylbenzene at 120 °C with the catalysis of Sn(Oct)_2_ or in bulk at 150 °C in the presence of ZnCl_2_ catalyst. Later, they transformed P3HB into the bicyclic ether-ester monomer 4-MOHB by combining ethyl 3-hydroxybutyrate and cyclohexene oxide (CHO) (Fig. 12a) [76]. Sn(Oct)_2_/BnOH catalyzed the ROP of 4-MOHB into P(4-MOHB), which can be selectively depolymerized into 4-MOHB in the presence of *p*-toluenesulfonic acid (TsOH) or Sn(Oct)_2_. Recently, they synthesized a series of enantiopure bicyclic ether-ester monomers with *trans*-fused cyclohexyl or cyclopentyl moieties, which underwent stereoretentive ROP in the presence of Sn(Oct)_2_ [77]. The resulting stereoregular polyesters (Fig. 12b) were crystalline, displaying high *T*_m_ up to 176 °C. These polyesters can be depolymerized back to their pristine monomers. More recently, they prepared a pair of enantiopure O-heterocyclic lactones bearing with pendent phenyl substituent, which are named as SR-M1 and RR-M2 (Fig. 12a). The well-controlled ROP of SR-M1 and RR-M2 were achieved by MeAl[salen] catalyst, producing poly(ether-ester)s with controlled molecular weight and narrow dispersity. Depolymerization of resulting polyesters back to pristine monomers in solution can be easily realized by using Sn(Oct)_2_ as a catalyst [78].

## 4 Chemical closed-loop recycling of polycarbonate

Polycarbonates have also received intense attention, due to their excellent biocompatibility and biodegradability similar to polyesters. The strategies for synthesis of polycarbonates are more diverse than that for polyesters, including ring-opening copolymerization (ROCOP) of CO_2_ and epoxides, ROP of cyclic carbonates, as well as polycondensation of diols and decarbonates. However, only the first two methods achieved the closed-loop life cycles.

### 4.1 Polycarbonates obtained from ROCOP

The ROCOP of CO_2_ and epoxides provides a powerful method in synthesizing polycarbonates, by taking advantage of high ring tension in epoxides to drive the reaction [79]. The properties of the resulting polycarbonates, such as thermal behavior, crystallinity, and hydrophilicity, can be tailored by varying the structure and functional groups of the epoxides. Moreover, the use of non-toxic and cost-effective CO_2_ make it become an economically viable option for synthesis of the environmentally friend copolymers. While the thermodynamically favored product obtained from the epoxide and CO_2_ coupling is a 5-membered cyclic carbonate, strategic selection of catalysts and polymerization conditions achieved high conversion rates and yields. However, the chemical recycling of CO_2_-derived polymers faces challenges due to the stability of cyclic carbonates. The genuine chemical recycling is to depolymerize the polycarbonates back into epoxides and CO_2_. It is reported that the degradation products derived from polycarbonates included *cis*-cyclic carbonates, *trans*-cyclic carbonates, epoxides, and CO_2_. Their distributions are significantly influenced by the structure, substituents and incorporated heteroatoms of the polymer, which would affect the cyclic behavior of the polycarbonates in turn.

**Fig. 13:**
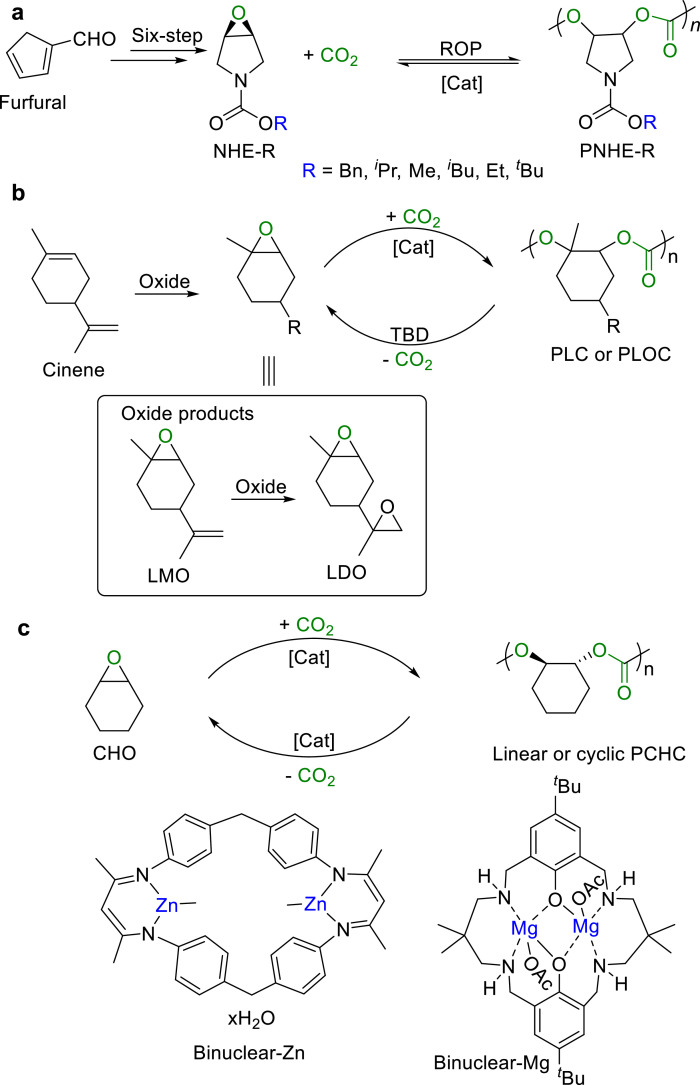
**Structure of the chemically recyclable (a) NHE-R monomers, (b) PLC and PLOC, (c) PCPC and the employed catalysts**.

In 2013, Darensbourg et al. reported the first chemical recycling example of polycarbonate that (salen)Cr^III^Cl/*^n^*Bu_4_NN_3_) catalyzed the depolymerization of poly(cyclopentene carbonate) (PCPC) into cyclopentene epoxide (CPO) and cyclic carbonate in 92% and 8% yields, respectively [80,81]. Later, Lu and Liu et al. prepared an epoxy monomer containing pyrrole ring by using the bio-based furfural as a reactant (Fig. 13a) [82,83]. ROCOP of such epoxy-alkanes with CO_2_ can be realized by [(salen)Cr^III^Cl]_2_/2PPNX (X = Cl^−^, F^−^, NO_3_^−^ or N_3_^−^) catalyst systems. The substituents of pyrrole ring can significantly influence the thermal stability and crystallization behavior of CO_2_-based polycarbonates. Polycarbonates with *^t^*Bu- substituents show high thermal stability (*T*_g_ up to 152 °C), similar to bisphenol A-based polycarbonates. Those with *^i^*Bu- substituents are stable up to ∼300 °C, while the Me- and Bn-substituted ones crystallize. Above 100 °C, the polycarbonates can be selectively depolymerized into epoxy-alkanes. Such depolymerization process can be repeated for many times by adjusting temperature, maintaining over 99% selectivity without formation of cyclic carbonate by-products.

In 2017, Sablong et al. employed TBD for depolymerization of OH-terminated poly(limonene carbonate) (PLC) or poly(limonene-8,9-oxide carbonate) (PLOC) (Fig. 13b) back to the epoxide monomers [84]. Recently, Zhang's group reported a binuclear zinc complex catalyzed copolymerization of CHO and CO_2_ into cyclic poly(cyclohexene carbonate) (PCHC), which can be depolymerized back to CHO in 99% selectivity by a polynuclear zinc compound (Fig. 13c) [85,86]. Concurrently, Buchard and Williams et al. utilized a bimetallic magnesium complex to realize the degradation of PCHC to CHO in 98% selectivity at 120 °C (Fig. 13c) [87]. With the catalysis of Salen-Cr^III^ and quaternary ammonium salt catalyst, Lu and Liu et al. achieved quantitative depolymerization of PCHC into CHO and CO_2_ at 140–200 °C.

### 4.2 Polycarbonates obtained from ROP

Typically, polycarbonates prepared by ROP requires six-membered rings and macrocyclic carbonate monomers [88]. Despite the structural similarities between the five-membered cyclic carbonate and the CO_2_-based polycarbonates produced from ROCOP of CO_2_ and epoxides, it is difficult to polymerize these five-membered monomers due to their low ring tension and high thermodynamic stability, often leading to decarboxylation. Six-membered cyclic carbonate monomers, prepared from the condensation of 1,3-diol substrates with solid phosgene sources, allowed the functional group diversity, which is beneficial for the regulation of the materials’ structures and functions [89]. These monomers show higher ROP activity than that of the five-membered ones, though complete conversion is still challenging due to equilibrium issues.

**Fig. 14:**
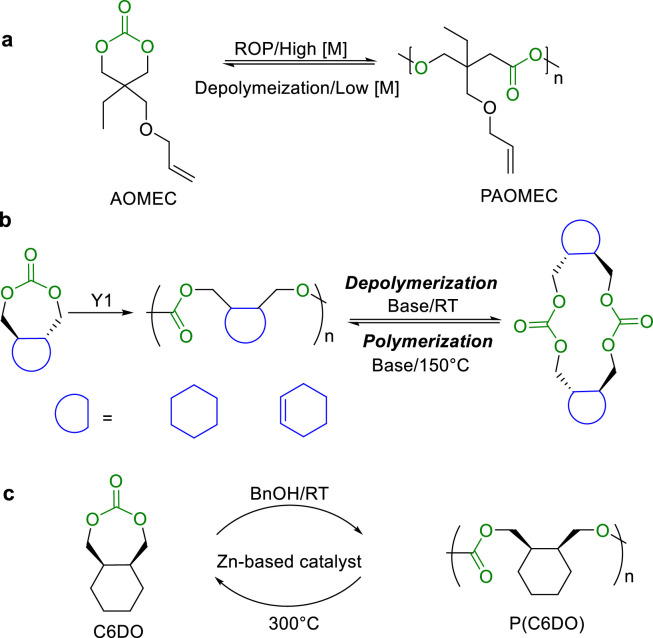
The chemical recyclable (a) PAOMEC and (b) seven-membered cyclic carbonates with *trans*-cyclic structures. (c) ROP of C6DO and chemical recycling to monomer.

Albertsson et al. employed 1,3-propylene glycol to synthesize a six-membered cyclic carbonate monomer, 2-allyloxymethyl-2-ethyl-trimethylene carbonate (AOMEC) (Fig. 14a) [90]. It is possible to control the polymerization and depolymerization thermodynamic equilibrium in the presence of DBU catalyst, by varying the reaction temperature, monomer concentration, and solvent polarity. However, the depolymerization is typically accompanied with some side reactions, such as decarboxylation, functional group decomposition, and oligomers formation. Odelius and co-workers developed macrocyclic carbonates (MCs) through selective ring-closure depolymerization of polycarbonates [88]. These MCs exhibited ultrafast polymerization rates and high conversion under mild conditions. An “odd-even” effect was observed in polymerization rates, which is dependent on the number of methylene groups between carbonate linkages, rather than the overall ring size. The resulting polymers can be chemically recycled into corresponding cyclic monomers.

Although the slightly higher ring tension of seven-membered cyclic carbonates makes them easier to be polymerized than the six-membered ones, it is challenging to synthesize seven-membered cyclic monomers. Zhu and Cai et al. designed and synthesized high-activity monomers, by introducing the *trans*-cyclic structures to the seven-membered cyclic carbonates (Fig. 14b), which enabled the production of high molecular weight aliphatic polycarbonates with low loading of metal yttrium catalysts [91]. MeOK catalyzed the selective depolymerization of these polycarbonates into macrocyclic carbonate dimers at room temperature, and the subsequent ROP at 150 °C offered a new way for the recycling of the high *T*_c_ monomers. More recently, they further developed a seven-membered cyclic carbonate (C6DO) with *cis*-fused cyclohexane structure (Fig. 14c). The ROP of C6DO produced aliphatic polycarbonate P(C6DO) with high molecular weight and narrow polydispersity, which could be effectively and selectively depolymerized into corresponding monomer C6DO, establishing a circular plastic economy [92].

## 5 Chemical closed-loop recycling of sulfur-containing polymers

Recently, intense interests have been focused on the closed-loop recycling of sulfur-containing polymers, due to larger Van der Waals radius of sulfur than oxygen (*R*_S_ = 1.80 Å vs *R*_O_ = 1.52 Å). This structural difference enhances the ring strain in thiolactones, favoring the ring-closing depolymerization and making thioesters more reactive towards the nucleophiles than esters. The sulfur's inclusion not only makes ROP and depolymerization under milder conditions easier but also imparts high refractive index, crystallinity, and degradation ability to the materials. This opened the door to the development of the engineering plastics, self-healing materials, and optical materials [93].

**Fig. 15:**
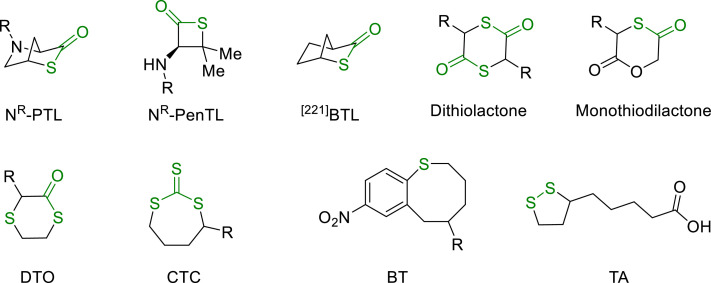
**Chemical recyclable sulfur-containing monomers**.

In 2019, Lu and co-workers reported the closed-loop recycling of sustainable polythioesters derived from 4-hydroxyproline (Fig. 15) [94]. Polythioesters were prepared with high molecular weight (*M*_n_ up to 259 kg/mol) and narrow molecular weight distribution (*Ð* < 1.15), with the catalysis of Et_3_N. Later, they reported the closed-loop recycling of penicillamine-derived β-thiolactones N^R^-PenTL via geminal disubstitution effect (Fig. 15) [95].

In 2020, Chen and Li et al. demonstrated that controlled polymerization and depolymerization of the bridged bicyclic thiolactone monomer, 2-thiabicyclo[2.2.1]heptan-3-one (^[221]^BTL), can be achieved by using a parallel ring strategy (Fig. 15) [96]. Tao et al. found that dithiolactone monomers are easier to be synthesized, exhibiting high kinetic polymerization activity and facilitating the closed-loop recovery [97,98]. A series of dithiolactone monomers were synthesized from the α-amino acids with different functional side groups (Fig. 15). In the presence of 4-dimethylaminopyridine (DMAP)/benzyl mercaptan, a series of polythioesters were synthesized with controlled molecular weight (*M*_n_ up to 101 kg/mol). The employment of DBU as a catalyst realized the closed-loop recycle of polythioesters within two minutes. Later, they also achieved the closed-loop recycle of monothiodilactones (Fig. 15) [99]. Due to the n→π* interaction between the adjacent ester and thioester in the polymer backbone, monothiodilactones showed high selectivity towards the propagation over transthioesterification.

In addition, Zhu and Cai et al. developed 1,4-dithian-2-one (DTO) with thioether and thioester functionalities (Fig. 15), which is similar with PDO monomer [100]. ROP of these DTOs showed excellent reactivity (TOF up to 2.3 × 104 h^-1^), affording poly(thioether-thioester)s with high crystallinity, and high-density polyethylene-like mechanical property (*σ*_B_ = 29.59 ± 1.08 MPa and *ε*_B_ = 749% ± 36%). Intriguingly, the chemical recycling of P(DTO) to monomer can be achieved under mild conditions within 1 min, showing circular life cycle. Ren's research group reported the ROP of recycled seven-membered cyclic trithiocarbonate δ-CTC and MCTC (Fig. 15) [101]. They found that using an organic base as a catalyst resulted in the formation of cyclic polymers, while utilizing BnSH as an initiator produced linear polymers. Both cyclic and linear polymers can be effectively recycled back to monomers upon heating, demonstrating excellent recyclability of seven-membered cyclic trithiocarbonates. Compared to its analogs, poly(trithiocarbonate) exhibited properties superior to that of poly(butanediol carbonate), including crystallinity, mechanical strength, and optical properties. Recently, Gutekunst et al. reported a chemically recyclable polythioether derived from benzothiocane (BT) monomers (Fig. 15) [102]. This system is based on the dynamic chemistry of nucleophilic aromatic substitution (S_N_Ar). Quantitative monomer conversion of benzothiocane can be reached in few minutes, furnishing materials with tailored properties simply by adjusting the functionalities of pendants.

Moreover, dynamic disulfide bonds have exhibited wide applications in the fields of covalent adaptive networks, polymer materials, etc. Thioctic acid (TA), a naturally occurring small molecule (Fig. 15), plays a vital role as a coenzyme in aerobic metabolism of animals. Tian and Qu et al. discovered that mild and complete depolymerization of TA-based polymers into monomers can be achieved in diluted alkaline aqueous solutions, furnishing recovered monomers in up to 86% yields [[103], [104], [105], [106], [107]]. Some newly designed, chemically recyclable TA-based polymers, such as the catechol-functionalized poly(disulfides) network, acylhydrazine-based reticular hydrogen bonds, dynamic supramolecular materials were reported recently [[108], [109], [110], [111], [112]]. In comparison with other sulfur-containing polymers, the TA-based materials are relatively easier to be prepared with a wide range of functionalities, which exhibited promising application potential in the closed-loop recycling of polymers.

## 6 Chemical closed-loop recycling of polar vinyl polymers and polyolefins

Polyolefins and polar vinyl polymers, being the major components of the today's plastic products, present challenges in terms of degradation and recycling due to their inert carbon-carbon bonds. High temperatures are typically required to overcome high energy barriers for cleavage of the C—C bonds. For instance, poly(methyl methacrylate) (PMMA) can be degraded at temperatures above 300 °C, albeit the low yields. Recently, remarkable progress has been made in the depolymerization of PMMA under mild conditions, thanks to the high end-group fidelity and functionality of certain PMMA derivatives [113,114].

**Fig. 16:**
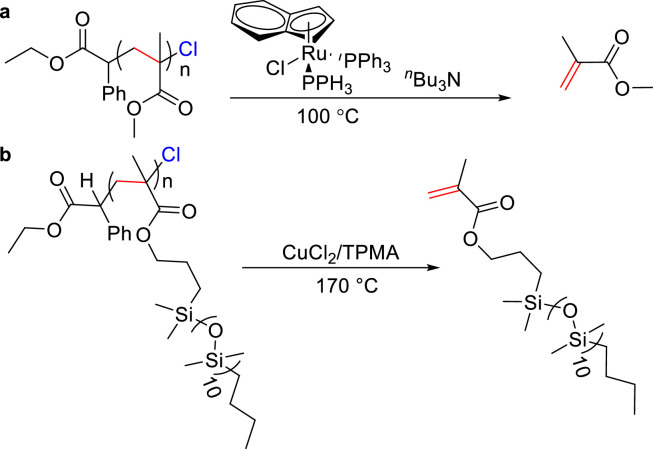
**Chemically recyclable (a) PMMA-Cl by Ru-based catalyst and (b) P(PDMSMA)-Cl by CuCl**_**2**_**/TPMA**.

Compared to other polymethacrylate macro-monomers, it is particularly challenging to depolymerize PMMA due to its higher enthalpy of polymerization, positive entropy, and higher repeat unit density. In 2019, Ouchi and co-workers employed a ruthenium catalyst (Ru(Ind)) to realize radical depolymerization of PMMA-Cl at 80 °C, specifically through the reversible activation of chlorine end-group (Fig. 16a) [115]. Although the depolymerization conversion rate was obtained in 4.5% to 6.6% by this method, it represented a significant step in the depolymerization of polymethacrylate produced by atom transfer radical polymerization (ATRP).

Matyjaszewski et al. also investigated the depolymerization of chlorine-capped poly(poly-(dimethylsiloxane)methacrylate)) (P(PDMSMA)), by using a CuCl_2_/tris(2-pyridylmethyl)-amine catalyst system (Fig. 16b) [116]. This approach afforded monomers in 80% yield at 170 °C, demonstrating the feasibility of depolymerization via radical formation through atom transfer. They utilized iron catalysts to further extend ATRP depolymerization to both the PMMA and poly(*n*‑butyl methacrylate) (PBMA). The reversible addition-fragmentation chain-transfer (RAFT) mechanism was adopted for the depolymerization of polymethacrylate.

**Fig. 17:**
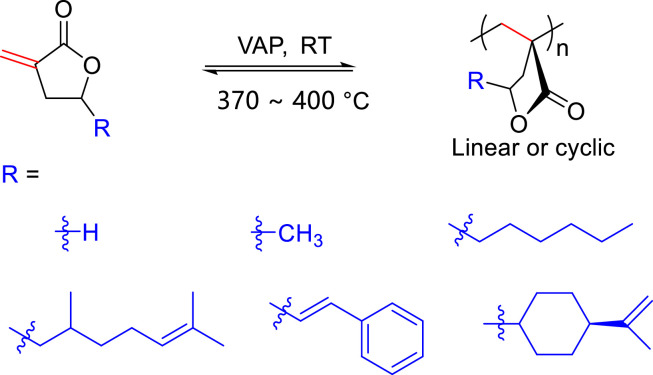
**Chemically recyclable PMMBL and its analogues**.

In 2020, Chen and co-workers explored the renewable alternatives to PMMA, such as poly(α-methylene-γ-butyrolactone) (PMBL) and poly(γ-methyl-α-methylene-γ-butyrolactone) (PMMBL), which can be selectively depolymerized back to the monomers in higher yields and purer form relative to PMMA [117]. PMBL and PMMBL also exhibited improved thermal stability and chemical recyclability (Fig. 17). Through theoretical calculation, they proposed that the enhanced recyclability of P(M)MBL bioplastics relative to PMMA is not resulted from their differences in *T*_c_ values, but the difference between the primary and tertiary macroradicals generated by linear ester and cyclic ester on the stability and monomer-production during random chain scissions. Later, Hong group reported the synthesis of different γ-substituted methylene butyrolactones from biomass (Fig. 17) [118]. These monomers can be polymerized in a controlled manner with the catalysis of Lewis pairs. The resulting polymers can be degraded into pristine monomers through thermolysis (up to 99.8% yield).

More recently, Zhang and co-workers reported a tethered B-P-B trifunctional, intramolecular frustrated Lewis pair catalyst for synthesis of an authentic cyclic PMMBL (*c*-PMMBL) [119]. They proposed an unprecedented chain initiation and propagation mechanism that pairwise monomer enchainment enabled by the cooperative and synergistic initiator/catalyst sites of the trifunctional catalyst. During polymerization, macrocyclic intermediates and transition states comprising two catalyst molecules were involved in the catalyst-regulated ring expansion and eventual cyclization, forming *c*-PMMBL and concurrently regenerating the catalyst. The *c*-PMMBL showed a ∼50 °C higher onset decomposition temperature and a much narrower degradation window than their linear counterparts. More importantly, *c*-PMMBL also maintained high chemical recyclability.

Aside from the carbon-carbon double bond-containing polymers, olefin polymers produced from the ROMP of cyclic olefins can also be designed for efficient recycling. It is reported that the optimization of monomer concentration and reaction temperatures can enhance monomer conversion for polymerization of cyclopentene and its derivatives as well as the monomer recovery yield for depolymerization of the corresponding polymers, respectively [120]. Introducing substituents to the cyclopentene ring further reduced ring tension and improved both the degradabilities and recyclabilities of resulting polymers.

**Fig. 18:**
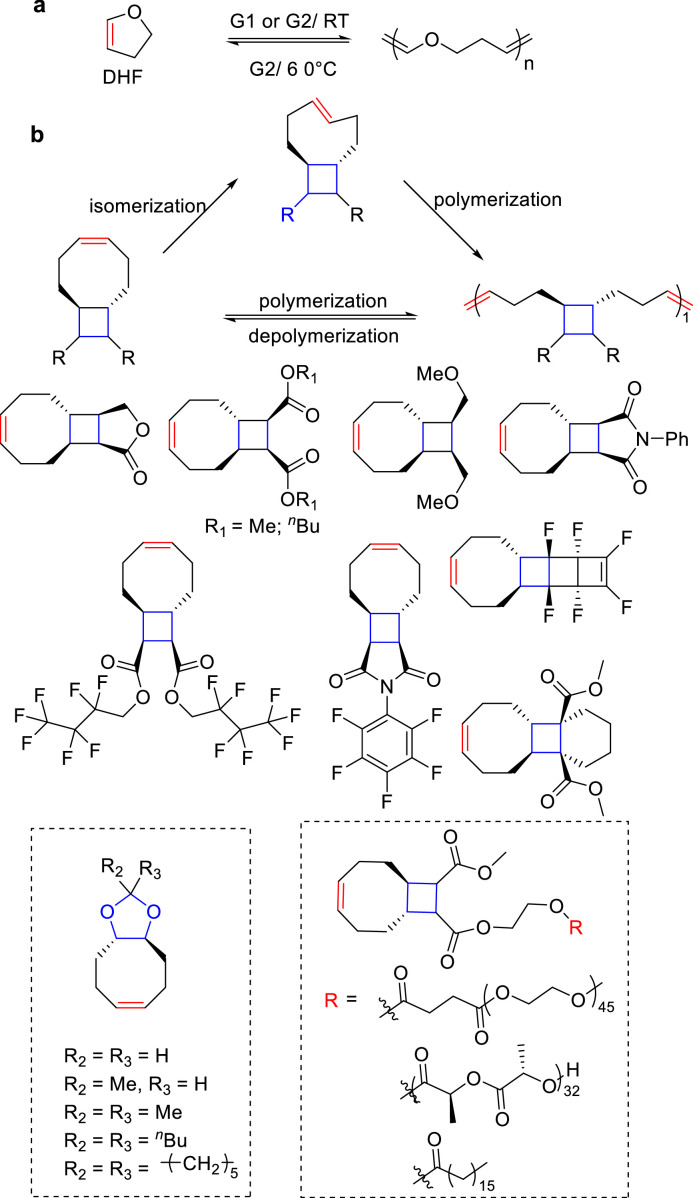
**Chemically recyclable (a) DHF and (b) cycloalkenes-based monomers**.

In 2020, Xia et al. investigated the ROMP of 2,3-dihydrofuran (DHF), by using a Grubbs Catalyst 2nd Generation (G2) or Grubbs Catalyst 1st Generation catalyst (G1) [121] (Fig. 18a). They demonstrated that the polymers can be efficiently depolymerized back to the monomers with high purity at 60 °C in the presence of G2 catalyst. Moreover, the DHF-based polymers can be decomposed into small molecular compounds under acidic conditions.

In order to explore the degradable polymers with better performance, Wang and co-workers studied the theoretical ring strain energies of different cycloalkenes (Fig. 18b), [122]. They found that the ring strain energies for cyclooctene is 8.2 kcal/mol, which is higher than that of cyclopentene (5.2 kcal/mol), therefore, the polymers cannot be depolymerized. However, the cyclooctene-based monomers with *trans* fused-ring exhibited similar ring strain energies with cyclopentene, and the *T*_g_s of corresponding polymers is above 100 °C. Upon heating these polymers at 50 °C in the presence of G2 (1mol%) in chloroform, up to 90% of polymers were depolymerized within 2 h. The thermal decomposition of these polymers only occurred above 350 °C. Progress was further made in the investigation of structure-polymerization thermodynamic relationships of the fused-ring cyclooctenes, substituent effects of the monomers, and preparation of a series of fused-ring cyclooctenes based polymers with different functionalities, which clearly demonstrates the application potential of ring-closing metathesis depolymerization as a low-energy depolymerization pathway [123,124].

**Fig. 19:**
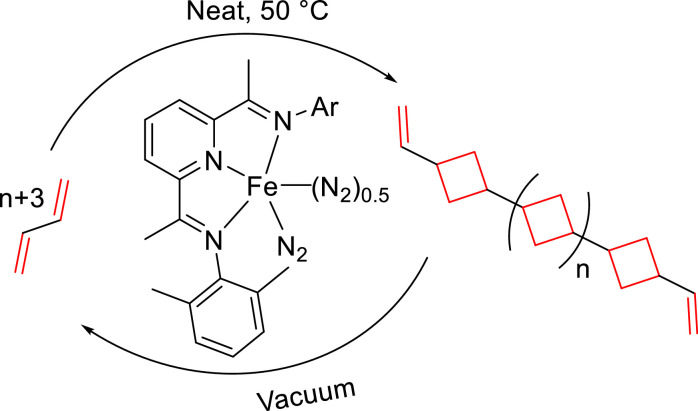
**Chemical recycling of 1,3-butadiene by Fe-based catalyst**.

Besides the above-described promising methods for chemical recycling of polymers, few examples have been applied to materials derived from abundant commodity olefinic monomers. In 2021, Chirik et al. reported a pyridine(diimine) iron catalyst for [2 + 2] cycloaddition/oligomerization of 1,3-butadiene (Fig. 19), furnishing a (1,*n*′-divinyl)oligocyclobutane polymers with carbon-carbon double bonds [125]. This polymer can be depolymerized back into pristine butadiene, showcasing the closed-loop chemical recycling of the hydrocarbon-based materials.

## 7 Chemical closed-loop recycling of other type polymers

**Fig. 20:**
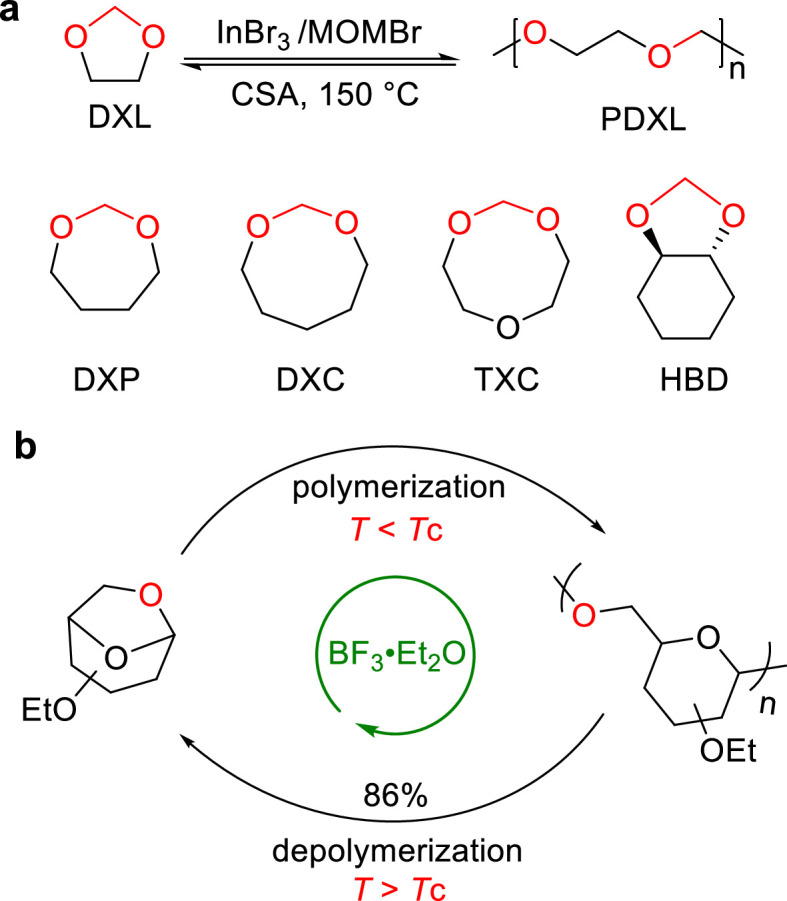
**Chemical recycling of (a) cyclic acetals and (b) 1,6-anhydrosugars**.

In addition to the above-mentioned recyclable polymers, there is ongoing research focused on the development of recyclable polymers with high-performance characteristics. Owing to their suitable thermal and chemical stability for practical applications, polyacetals can serve as promising candidates for chemical recycling as it is capable of being depolymerized at low temperatures (< 150 °C), probably due to the presence of the dynamic acetal group. However, it is challenging to synthesize high molecular weight polyacetals from the cyclic acetal monomers. By using InBr_3_ and MOMBr as a catalyst, Coates and co-workers reported a reversible-deactivation cationic ROP of cyclic acetals (Fig. 20a) [126]. The resulting poly(1,3-dioxolane) (PDXL) exhibited mechanical properties (σ_B_ = 40.4 ± 1.2 MPa and ɛ_Β_ = 720 ± 20%) comparable to those of HDPE and LLDPE. More importantly, PDXL can be depolymerized back into DXL monomer in 98% excellent yield, with the catalysis of 2% camphorsulfonic acid. Later, they achieved ultra-high-molecular-weight (UHMW) PDXL with *M*_n_ value up to 2031 kg/mol, by using [Et_3_O^+^][PF_6_^−^] as a catalyst [127]. This UHMW PDXL exhibited tensile properties similar to UHMW polyethylene, demonstrating promising potential of chemically recyclable UHMW PDXL in replacing the high-performance thermoplastic polyolefin materials.

Recently, Niu and co-workers reported a chemical method for precise synthesis of polysaccharides through the BF_3_-catalyzed living cationic ROP of 1,6-anhydrosugars, including d-glucan, d-mannan, and l-glucan (Fig. 20b) [128]. Despite these polysaccharides exhibiting high *T*_m_ value (up to 284 °C), they can be completely depolymerized back into monomers in impressive yields (up to 86%) at 80 °C, in the presence of 5 mol% BF_3_·Et_2_O.

## 8 Perspective and outlooks

In summary, we have reviewed the recent examples that demonstrate the capability of rational monomer design strategy to strike fine balance between the conflicting properties such as polymerizability, recyclability, and performance. Through the intelligent design of monomer structures and regulation of thermodynamic and kinetic factors in polymerization, completely chemical recyclability can be achieved within polymerization systems. However, several unresolved challenges are still associated with this emerging strategy:

First and foremost, further investigation is needed for better understanding of the intricate relationship between the monomer structures, polymerization activities, polymer properties, and depolymerization. Theoretical and computational studies will play a vital role in high throughput exploration of novel monomer structures and development of advanced polymer properties. It is crucially important for making advancements in this field to establish clear structure-performance relationships and achieve a seamless integration of these factors.

Second, the designed, recyclable monomers should be derived from a broad range of sources, preferably from renewable resources. Moreover, these monomers should be prepared in a straightforward and efficient way, ensuring these monomers with enough competitiveness and sustainability as excellent alternatives to the non-renewable polymers.

Third, it is important to develop innovative approaches for highly selective depolymerization of polymers blends under mild conditions. Real-world applications often involve the mixture of multiple materials rather than a single material. Therefore, it is on the must-to-do list for polymer chemists.

Addressing these challenges will definitely facilitate the development of the rational monomer design strategy in synthesizing recyclable and high-performance polymers.

## Declaration of competing interest

The authors declare that they have no conflicts of interest in this work.
